# “To Normalize is to Impose a Requirement on an Existence.” Why Health Professionals Should Think Twice Before Using the Term “Normal” With Patients

**DOI:** 10.1007/s11673-021-10122-2

**Published:** 2021-10-18

**Authors:** Michael Rost

**Affiliations:** Institute for Biomedical Ethics, University of Basel, Switzerland, Bernoullistr. 28, 4056, Basel, Switzerland

**Keywords:** Normality, Normal, Discrimination, Is-Ought, Statistics, Normativity

## Abstract

The term “normal” is culturally ubiquitous and conceptually vague. Interestingly, it appears to be a descriptive-normative-hybrid which, unnoticedly, bridges the gap between the descriptive and the normative. People’s beliefs about normality are descriptive and prescriptive and depend on both an average and an ideal. Besides, the term has generally garnered popularity in medicine. However, if medicine heavily relies on the normal, then it should point out how it relates to the concept of health or to statistics, and what, after all, normal means. Most importantly, the normativity of the normal needs to be addressed. Since the apparently neutral label “normal” can exclude, stigmatize, and marginalize people who are defined in contrast to it as abnormal, health professionals should think twice before using the term with patients. The present critical perspective advocates against using the term “normal,” as long as no understanding of a person’s individual normality has been attained. It advocates for the right to autonomously determine one’s own normality. For health professionals I do not see worthwhile benefits of subscribing to the concept of (non-individual and normatively loaded) “normality” and imposing it on their patients. But I do see many risks.

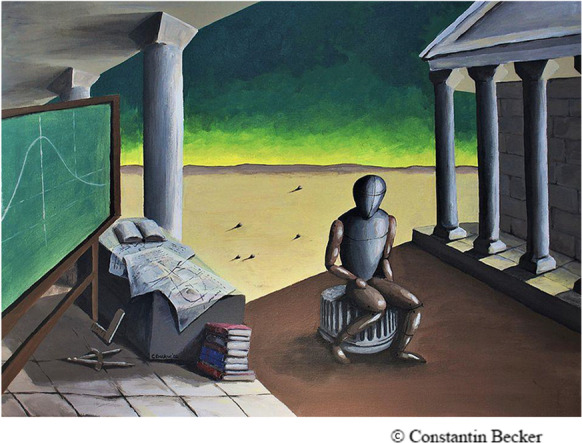

“Normal” is a strange word. We use it constantly, like our favourite mug, but our understanding of it remains volatile, like the scent of the freshly brewed coffee in our mug. In the following, I will critically discuss the subject of normality with the aim to make people think about the normal, its fallaciousness, and its dangers.

## “You want to watch out for [words] whose influence is felt everywhere, but whose location and operation remain somehow invisible.” (Stephens 2019, 278)

The term “normal” is culturally ubiquitous, but its conceptual essence has not been fully unveiled (Stephens 2019). If a concept is widely used among both lay persons and health professionals, one might expect a well-elaborated understanding of what it means. Unsurprisingly, this assumption is not met in the case of the normal, which is dynamic and contingent on cultural and historical circumstances. To make things even worse, multiple domain-specific normalities coexist.

In times of crisis, typically an uptick in prevalence of the term “normal” can be observed. Just compare pre-pandemic and pandemic numbers or use Google-Books-Ngram-Viewer to see the increase during and especially after political crises. In these cases, the term “normal” is mostly applied to social, political, and economic conditions. This should not obscure another field of application, namely persons and their qualities.

The present opinion piece addresses this particular field of application. As the coronavirus spreads, the use of the term “normal”—primarily being applied to social conditions, such as a return to normality as indicated by an end of curfews—spreads as well, and this risks to reinforce the acceptance of the concept of normality, also when applied to persons. This is unfortunate because, as will be shown later, the label “normal” often stigmatizes, marginalizes, and sometimes even pathologizes persons who are not considered normal.

## The normal “uses a power as old as Aristotle to bridge the fact/value distinction, whispering in your ear that what is normal is also right” (Hacking 1990, 160)

Ethical analysis distinguishes between two types of statements. Descriptive statements make factual claims about how the world or a person is. Normative statements make prescriptive claims about how the world or a person ought to be. Normative conclusions (e.g. how persons should behave, which qualities are good) need to rest on at least one normative premise. Of course, as with descriptive premises, the normative premise is always debatable, but the point is that an ought (i.e. normative claim) cannot solely rest on an is (descriptive claim).

Interestingly, the normal appears to be a descriptive-normative-hybrid. It seems to unnoticedly bridge the gap between the descriptive and the normative, a gap which philosophers have been struggling with for eons. In “The Normal and the Pathological,” Canguilhem articulates this convergence: “the concept of normal is itself normative” (Canguilhem 1991, 241). Correspondingly, recent psychological research evidences that “people actively combine statistical and prescriptive information [i.e. descriptive and normative statements] ( … ) into an undifferentiated notion of what is normal” (Bear and Knobe 2017, 25; Wysocki 2020). People’s beliefs about normality, thus, are descriptive (i.e. average, frequency) and prescriptive (i.e. ideal, goodness) and extend beyond a mere description of persons into the realm of moral norms.

Yet, where does normality’s normative force come from? Most of the time, normality is derived from statistics (e.g. averages), which, by nature, represent mere descriptive statements about the distribution of measured qualities and which lack any intrinsic normative significance. For the normal distribution, normal refers to the situation where most of the sample data clusters around a single value (the mean) with observations far apart from this value being rare, but it neither describes one part of a binary normal-abnormal condition (Cryle and Stephens 2017) nor defines some sort of statistical normativity. Here, human qualities follow a normal distribution and every manifestation is normal. In themselves, such descriptive statements cannot be translated into normative claims. Statistics are immensely important for science, but they are “detrimental when used as a blunt instrument of measurement to legitimize labels [e.g. normal] that differentially sort people into subpopulations that augment social inequalities” (Mason 2015, 343). Alas, despite lacking a normative underpinning, the normal usually embraces a non-justified ought, which comes along perfectly disguised as a well-justified moral norm.

## “Normality is a term which recurs with disturbing frequency in the writings of psychologists, psychiatrists, psychoanalysts, sociologists” (Eysenck 1953, 177)

The term “normal” has generally garnered popularity in medicine. A brief look at the occurrences within the major classification systems not only reveals its importance but also provides first hints as to where the normal unfolds its normative power (Table 1). While personalized medicine’s focus on individual characteristics of patients might one day erode the significance of normality in medicine (Chadwick 2017), today, undoubtedly, the normal is a major diagnostic category in medicine. However, these diagnostic classification systems no longer consider the entirety of manifestations of human qualities as normal. Instead, limits to normality are set and cut off from both tails of a distribution which is mathematically infinite. Apparently, medicine “reserves the right to confer labels of normality and abnormality, but to what extent are these terms objective and purely descriptive?” (Mason 2015, 345)

**Table 1:** Frequencies^a^ and examples of the term “normal” within classification systems

	Frequency	Examples
**DSM-V**^b^	366 instances 1x/2.7 pages	“normal life variation,” “abnormalities of emotional or cognitive processing,” “normal fluency of speech,” “normal developmental variations,” “normal sexual desire,” “normal pattern of learning academic skills,” “abnormal social approach,” “normal level of intellectual functioning,” “abnormality of emotional processing”
**ICD-10**^c^	259 instances 1x/1.0 pages	“abnormalities of behaviour,” “normal social inhibitions,” “abnormal mood states,” “normal family relationships,” “normal sense of (fe)maleness,” “normal children”
**ICD-11**	1445 instances 1x/1.2 pages	“abnormal social behaviour,” “normal personality characteristics,” “normal delivery,” “normal range of life experiences,” “normal skin,” “normal grief,”, “normal speech,” “normal menopause”

^a^Author’s own counting;

^b^In comparison, within DSM-I 19 instances (1x/7.6 pages);

^c^Classification of Mental and Behavioural Disorders—Clinical descriptions and diagnostic guidelines.

Roughly, health has been conceptualized as an “objective notion,” determined by empirically observable symptoms (e.g. value-free biomedical model, corresponding to some sort of statistical normality) or as an “subjective notion,” socially and normatively constructed (e.g. value-laden sociopolitical model, corresponding to some sort of normative normality). Irrespective of this genuine contestability of the concept of health and the underlying notions of normality, if medicine heavily relies on the normal, then it should point out how the normal relates to the concept of health or to statistics, and what, after all, normal means. Reflection on the normal should be incorporated in classification systems and in medical curricula. The normal should be an object of critique. Its determination should not be left to the diagnosing health professional. Most importantly, the normativity of the normal needs to be addressed. However, normality in medicine is currently not clearly defined in the medical literature (Chadwick 2017; Catita, Águas, and Morgado 2020). Only if medicine achieves a shared operational conceptualization of normality and the source of normality’s normativity is expounded, the term “normal” could be used that widely in medicine.

## “This unassuming word can have a significant effect on the lives of those defined in contrast to it as abnormal, pathological, or deviant.” (Cryle and Stephens 2017, 2)

Normality, once established, is rarely made explicit but still powerfully permeates our daily lives. Any person-related reference to normality, simultaneously, qualifies abnormality. Facing this ineluctable truth, an individual might experience fear of denormalization, which often results in marginalization and stigmatization, and, hence, has strong incentives to adhere to the normal. In fact, any form of normality entails conformity pressure. “Normal” is no neutral label. On the contrary, it is—in its negative form: the abnormal—being used in a variety of discourses as a metonym for social exclusion. The normal and abnormal are powerful tools in the hands of those who construe its essence.

“Normal” is a generic replacement term that, mediated through persisting related beliefs, discriminates against certain—often historically excluded—groups in various mutually constitutive discourses. These discourses are interconnected and, therefore, discrimination at the intersections can be amplified (Crenshaw 1989). Intersectional groups can experience unique forms of overlapping discriminations due to multiple categorizations as abnormal. In this vein, the category of the abnormal can be understood as the nucleus of various forms of discrimination. It marks the area of densest overlap of discrimination fields. This is why the malleable normal is so dangerous. It feeds and, hence, spans many (medical) discourses and does not exclusively unfold its power in one separate field but across various fields. Many normalities exist and they bolster one another.

This is illustrated by the following few findings on the normative and exclusionary operation of normality that are related to medicine. The normal implicitly determines social judgements about the acceptability of certain kinds of biological variation (Amundson 2000). The bodily integrity of intersex people is threatened by the idea of normal sexes (i.e. binary) and by characterizing intersexed bodies as abnormal (Reis 2009). The idea of normal abilities imposes normative assumptions on persons with physical disabilities (Davis 1995). Equating psychological normality and mental disorders gives rise to questionable diagnostic labels (Bartlett 2011). The idea of a normal body brings about oppressive narratives about physically impaired people (Thomson 1997) and abets gendered norms about bodily appearance and bodily normalcy (Liebelt 2019; Kittay 2006). The construct of normal infant growth is used to force assimilation of indigenous people into the nation-state (Butt 1999). The myth of a normal brain leads to a pathologization and (dis)qualification of individual human brains as abnormal and ultimately disregards the notion of neurodiversity (Armstrong 2015).

**Fig. 1:**
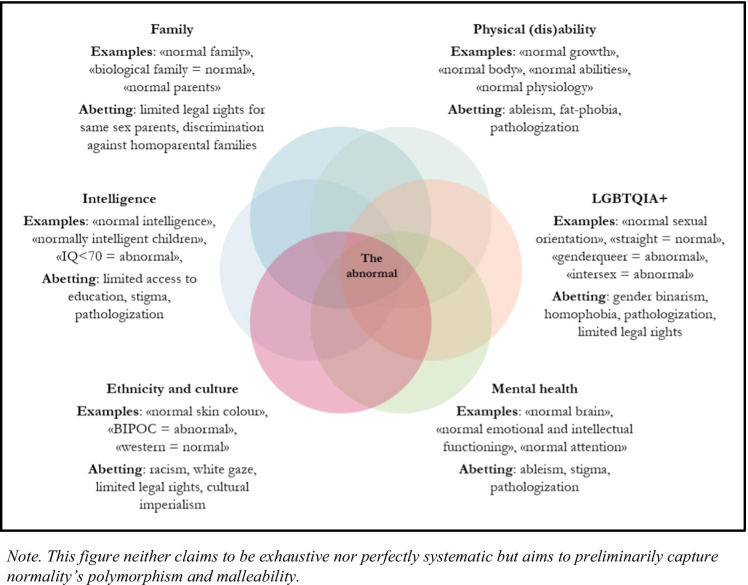
The normal in various discourses

Given these effects of the normal, shouldn’t we allow only one form of normality, that is each person’s individual normality? With respect to health, Goldstein outlined that “disease can be determined only by means of a norm which permits taking the entire concrete individuality into consideration, a norm which takes the individual himself as the measure; in other words, as an individual, personal norm” (Goldstein 1939, 433). Applying his health-related rationale to the determination of normality, ultimately, helped people to be who they are and to do what they value doing. In contrast, imposing (non-individual) normality on them unduly interferes with their flourishing. Related to this, it has to be realized that the term “normal” either refers to all human qualities and, therefore, applying it to persons becomes meaningless or it exclusively refers to a subset of human qualities and, thereby, excludes some from being normal (Fig. 1).

## “The benign and sterile-sounding word ‘normal’ has become one of the most powerful ideological tools of the twentieth [and twenty-first] century” (Hacking 1990, 169)

I am advocating against using the term “normal” with persons, particularly in medicine, as long as no understanding of their individual normality has been attained. I am advocating for the right to autonomously determine one’s own normality. No one should be subject to an imposition of normality. In short, normality should be determined intra- not inter-individually, internally not externally. Lacking an understanding of a person’s individual normality and still applying the term to the individual means to “impose a requirement on an existence” (Canguilhem 1991, 239). This is likely to cause harm on the part of the individual and violates the bioethical principle of primum-non-nocere. Refraining from using the term “normal” in the medical setting also means to undermine normatively loaded normality at a societal level. Without justificatory grounds it tells us what to do and what to be and, thereby, perpetuates systems of power, privilege, and inequality. These systems require efforts to preserve them, mainly on the part of the privileged and powerful. Taking away the(ir) normal helps to dismantle their hegemony.

Although historians studying normality are “unpersuaded (…) that the concept of normal relies on logical coherence, and that exposing its contradictions will fatally undermine its functionality” (Cryle and Stephens 2017, 9), you can still emphatically ask yourself, what would be lost, if you simply stopped using the term “normal” when referring to persons, refrained from using the term with patients? Can’t you always replace the term with a more accurate one? Do you really need it?

Regarding patient care, medical ethics frequently invokes a risk-benefit evaluation. To conclude, a patient, without any doubt, is free to define its own normality, but for health professionals I do not see worthwhile benefits of subscribing to the concept of (non-individual and normatively loaded) “normality” and imposing it on their patients. But I do see many risks. Medicine’s persistent recourse to normality not only—in many instances—fails to honour the paradigm of rigorous science, it also—more importantly—fails to honour the lived experiences of those systematically excluded by the social, cultural, and medical authority of the normal.
