# Correction to: “To Normalize is to Impose a Requirement on an Existence.” Why Health Professionals Should Think Twice Before Using the Term “Normal” With Patients

**DOI:** 10.1007/s11673-021-10152-w

**Published:** 2021-12-23

**Authors:** Michael Rost

**Affiliations:** Institute for Biomedical Ethics, University of Basel, Switzerland, Bernoullistr. 28, 4056 Basel, Switzerland

**Correction to: Bioethical Inquiry (2021) 18:389–394**

10.1007/s11673-021-10122-2

The figure in the opening page of this article was inadvertently published without mention of the artist's copyright. It should have read '© Constantin Becker' below the figure. The original article has been corrected.

